# An ancient process in a modern mollusc: early development of the shell in *Lymnaea stagnalis*

**DOI:** 10.1186/1471-213X-13-27

**Published:** 2013-07-12

**Authors:** Jennifer Hohagen, Daniel J Jackson

**Affiliations:** 1 Courant Research Centre Geobiology, Georg-August University of Göttingen, Goldschmidtstrasse 3, 37077, Göttingen, Germany

**Keywords:** Shell, Mollusc, Biomineralisation, Evolution, Development, Specification, Mantle, Alkaline phosphatase, Peroxidase

## Abstract

**Background:**

The morphological variety displayed by the molluscan shell underlies much of the evolutionary success of this phylum. However, the broad diversity of shell forms, sizes, ornamentations and functions contrasts with a deep conservation of early cell movements associated with the initiation of shell construction. This process begins during early embryogenesis with a thickening of an ectodermal, ‘dorsal’ (opposite the blastopore) population of cells, which then invaginates into the blastocoel to form the shell gland. The shell gland evaginates to form the shell field, which then expands and further differentiates to eventually become the adult shell-secreting organ commonly known as the mantle. Despite the deep conservation of the early shell forming developmental program across molluscan classes, little is known about the fine-scale cellular or molecular processes that underlie molluscan shell development.

**Results:**

Using modern imaging techniques we provide here a description of the morphogenesis of a gastropod shell gland and shell field using the pulmonate gastropod *Lymnaea stagnalis* as a model. We find supporting evidence for a hypothesis of molluscan shell gland specification proposed over 60 years ago, and present histochemical assays that can be used to identify a variety of larval shell stages and distinct cell populations in whole mounts.

**Conclusions:**

By providing a detailed spatial and temporal map of cell movements and differentiation events during early shell development in *L. stagnalis* we have established a platform for future work aimed at elucidation of the molecular mechanisms and regulatory networks that underlie the evo-devo of the molluscan shell.

## Background

Molluscs constitute one of the most successful, morphologically diverse and ancient phyla of the animal kingdom. They posses an extensive fossil record dating back to the early Cambrian (543+ MYA) and comprise more than 200,000 extant species occupying various marine and terrestrial environments from the deep sea to desert habitats [1,2]. Much of this evolutionary success can be attributed to the phenotypic plasticity of the external shell which displays an incredible range of mineralogical textures [3], pigments [4,5] and ornamentations [6]. This phenotypic diversity is underscored by a diversity in the molecular mechanisms responsible for the construction of the adult shell [7-10].

Despite the morphological and functional-molecular diversity of the adult shell, there is deep conservation of the cellular and morphogenic movements that initiate larval shell secretion (reviewed in [11]). Importantly, larval shell forming cells are thought to give rise to the fully differentiated adult shell forming organ, the mantle, suggesting that trochophore, veliger and adult gastropod shells do not have independent evolutionary origins as previously suggested [12]. Cell lineage studies in disparate gastropods support a common ontogenetic origin of embryonic, larval and adult gastropod shells; derivatives of the 2d and 2c micromeres in *Ilyanassa* give rise to the shell gland [13], and the same lineage of cells in *Crepidula fornicata* contributes to the mantle of the veliger [14]. Furthermore, veliger mantle cells expressing shell forming genes continue to do so following metamorphosis in the abalone *Haliotis asinina*[15,16]. The histochemical properties of larval and adult shell forming organs in *L. stagnalis* also reveal a similar spatial arrangement of enzymatic activities, suggesting that boundaries of shell forming cell populations established in larval stages are maintained into adult life [17]. Additionally, regulatory genes encoding transcription factors and signalling molecules (such as members of the Hox cluster, *engrailed* and *decapentaplegic*) are expressed in embryonic shell forming tissue in disparate molluscan taxa [18-25]. This raises the possibility that extant shelled molluscs may all initiate shell formation using the same developmental program inherited from a distant ancestor, and that it is the downstream shell forming programs operating in the mature mantle which, during evolution, have generated today’s diversity of shelled adult molluscs. If such a scenario were true, this would mean that a common ancestor of the shelled molluscs evolved a developmental program to form a shell which was passed on to all of its future descendants; a 540+ million year old innovation that was of great importance to the future evolutionary success of the phylum.

The pulmonate gastropod *Lymnaea stagnalis* (Linnaeus, 1758) was once a much used model for understanding both molluscan development in general [26] and development of the shell in particular [27]. Development of *Lymnaea’s* shell displays many of the features observed in other gastropod species. Across Molluscan classes, the first morphological sign of shell development is a thickening of the dorsal ectoderm in the post-trochal region of the embryo following gastrulation (see [11] for a review). Briefly, these dorsal ectodermal cells elongate and are often the only ectodermal cells in contact with the underlying endoderm, specifically cells at the tip of the archenteron (the so called 'small-celled endoderm' due to their lack of large vacuoles present in other endodermal cells [26,28]). These elongated dorsal ectodermal cells then invaginate to form a ‘shell gland’ [11,29]. It is during this stage that secretion of the first shell-associated insoluble material takes place. The shell gland subsequently evaginates to form the ‘shell field’, a process during which the contact of ectodermal and endodermal cells is lost, and the first signs of calcification of the previously secreted insoluble material can be observed (e.g. [13,26,27,30]). The initial contact between endoderm and dorsal ectoderm that precedes shell gland invagination has been observed in representatives of the Gastropoda, Bivalvia, Scaphopoda and Cephalopoda (reviewed in [11]). This contact between dorsal ectoderm and endoderm has lead to the idea that this event is required for the specification of future shell forming cells, and represents a 'true' induction event [26].

While Raven’s model of shell gland induction [26] represents the canonical theory of molluscan shell field specification, the molecular mechanisms that initiate and underlie this process remain largely unknown. Molecular analyses that have previously identified transcription factors and signalling molecules in the shell gland and the evaginated and expanding shell field are expressed well after the specification of shell forming cells [19,21,24,31]. We are therefore developing *L. stagnalis* as a model for molecular investigations into the mechanisms that first specify shell forming cells, and through comparative studies, to enhance our understanding of how the variety of molluscan shells evolved.

Previous cytological studies on the early development of the shell field in *L. stagnalis* do not include descriptions of the cellular arrangements preceding the first contact between the dorsal ectoderm and the small-celled endoderm at the tip of the archenteron. Here we employ confocal laser scanning microscopy (CLSM) to provide a detailed temporal and spatial description of the morphogenic events associated with development of the larval shell in *L. stagnalis*. We have also employed histochemical assays based on endogenous peroxidase (PO) and alkaline phosphatase (AP) activity to identify distinct cell populations within the developing shell gland, shell field and other larval structures in whole mounts. These enzymes are known to be active in the shell forming tissues of several molluscan taxa, including *L. stagnalis*[17]. These assays allow us to trace discrete cell populations in larval shell forming tissues, and may in the future be employed to characterise the effects of experiments aimed at the perturbation of normal shell development. This work represents a platform from which further studies will investigate the molecular processes leading to the specification and differentiation of molluscan shell forming cells.

## Methods

### Cultivation of adult *L. stagnalis*

Adult specimens of *L. stagnalis* were collected from the Northeimer Seenplatte near Northeim, Germany (51° 43’ 26.5368’, 9° 57’ 24.75’) and from a pond on the North campus of the University of Göttingen, Germany (51° 33’ 23.727’, 9° 57’ 25.617’). Snails were kept in standard tap water at 25°C, under a 16:8 light dark regime and fed *adlibidum* with lettuce and a variety of other vegetables.

### Staging and preparation of embryos of *L. stagnalis*

Freshly deposited egg masses were collected and their development monitored. Following the first cleavage, egg masses were cultured in snail water [32] at 25°C. All stages are indicated in hours post first cleavage (hpfc) and days post first cleavage (dpfc). At the desired developmental time point individual egg capsules were removed from an egg mass and freed from the jelly by rolling them over moist filter paper. Embryos were manually dissected from their capsules using forceps and needles and fixed according to the subsequent experimental procedure (see below).

### Confocal laser scanning microscopy (CLSM)

29 embryonic stages between 27 and 87 hpfc were fixed at intervals of one to five hours. For each developmental stage, 29 to 126 individuals were visualised, and on average 6 individuals were imaged. To account for fixation artefacts several fixation treatments were tested on embryos between 27 and 37 hpfc, ranging from no fixation to extended fixations overnight at room temperature and in varying amounts of gluteraldehyde in combination with a paraformaldehyde-based fixation. Fixation with 4% paraformaldehyde (PFA) in 1X phosphate buffered saline (PBS) for one hour at room temperature, or overnight at 4°C, was found to be optimal. Fixed specimens were washed three times in PBS and processed immediately or stored at 4°C for up to five weeks. For cytoplasmic and nuclear staining samples were incubated in a 1/1000 dilution of Sytox Orange (Molecular probes, S11368) in PBS with 0.1% TritonX for two hours at room temperature. Samples were then washed three times in PBS, dehydrated through a graded ethanol series and embedded in a 1:2 mixture of benzyl benzoate and benzyl alcohol (BB:BA). Optical sections were captured using a Zeiss LSM 510 Meta with the following settings: HeNe 543 laser at a power of 2.9%; pinhole between 50 μm and 60 μm (0.94 to 1.13 Airy Units); amplifier gain of 1; amplifier offset and gain adjusted to the sample brightness; stack size 1024 x 1024 with a stack thickness between 0.81 μm and 0.9 μm; scan speed and number of scans 7 and 4 or 6 and 8 respectively. For individual images the stack size was 2048 x 2048 with a scan speed and number of 6 and 8 respectively. All images were false-coloured and adjusted for brightness using Macnification version 2.0.1.

### Scanning electron microscopy

Between 55 and 278 embryos for each time point from 27 to 67 hpfc at five hour intervals were fixed in 2.5% gluteraldehyde in PBS at 4°C overnight. These were then dehydrated through a graded ethanol series and dried overnight in hexamethyldisilazane. Samples were mounted on carbon pads on aluminium stubs and sputter-coated with a gold-palladium alloy before being imaged with a scanning electron microscope at 3.8 kV. All SEM images were edited in Adobe Photoshop CS3 version 10.0.1 by applying the ‘auto levels’ and ‘auto contrast’ functions.

### Histology and Histochemistry

The endogenous alkaline phosphatase (AP) activity of 13 developmental stages ranging from 37 to 117 hpfc at 3 to 5 hour intervals was examined. Between 34 and 154 individuals were included in each experiment. Additionally, 14 older larvae (ranging from five days post first cleavage until hatching from the egg capsule) were also assayed for AP activity. For each developmental stage, images of three to 19 individuals were captured. Embryos were fixed for 45 to 60 min in 4% PFA in 1X PBS containing 0.1% Tween20 (PBTw), and rinsed in 1X PBS before being incubated in AP reaction buffer (100 mM Tris, 100 mM NaCl, pH 9.5) for 5 to 20 min. AP reaction buffer was replaced by detection buffer (AP reaction buffer, 50 mM MgCl_2_, 175 μg/mL BCIP and 450 μg/mL NBT). The colour reaction was stopped after 15 to 60 min at an optimal signal to background ratio by replacing the detection buffer with 0.1 M Glycine pH 2 containing 0.1% Tween20. Samples were then rinsed in PBS and post-fixed overnight at room temperature in 4% PFA in PBS, dehydrated through a graded ethanol series, embedded in BB:BA and viewed and photographed using a Zeiss microscope Axio Imager Z1. A fraction of larger, older (5+ dpfc) larvae were washed twice in PBTw following fixation, and then embedded in 60% glycerol and imaged under a Zeiss stereo microscope discovery V8.

For developmental stages between 47 hpfc and 5+ dpfc, the endogenous peroxidase (PO) activity of 45 to 139 individuals was examined prior to performing the AP assay (described above) in order to visualise the activity of both enzymes at once. Samples fixed as described above were first rinsed twice in 50 mM Tris pH 7.3, and then pre-incubated in 50 mM Tris pH 7.3 containing 1 mg/mL diaminobenzidine (DAB) for 15 to 20 min before supplementing the solution with a 1/3000 dilution of 30% hydrogen peroxide. The colour reaction was monitored and stopped (usually after one to two minutes) by rinsing the samples for about 10 min in 1X PBS. The AP assay (as described above) was then performed on this material. One to 13 individuals per developmental stage were photo-documented.

## Results

**Figure 1:**
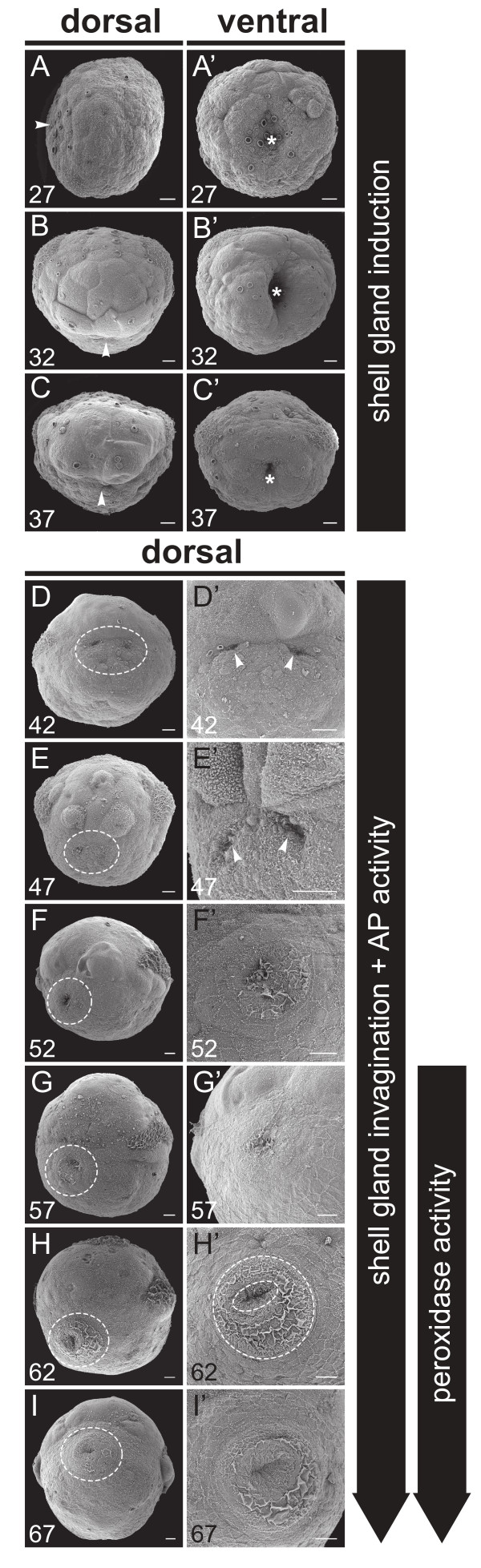
**Early shell development in *L. stagnalis *illustrated by Scanning Electron Microscopy (SEM). A**-**C’** Gastrulation and formation of the archenteron. The site of the future shell gland is marked by white arrowheads. The blastopore is marked by an asterisk. **D**-**E’** The first outward signs of shell gland invagination are two shallow depressions at 42 hpfc (arrowheads in **D’** and **E’**). **F**-**I’** Insoluble material secreted by the shell gland is visible from 52 hpfc onwards. The first asymmetry of the shell is evident at 62 hpfc (highlighted by two dashed ovals in H’). All scale bars are 10 μm. Numbers in the lower left of each panel indicate the age in hours post first cleavage (hpfc). Panel G is reflected about the vertical axis for consistency of presentation.

**Figure 2:**
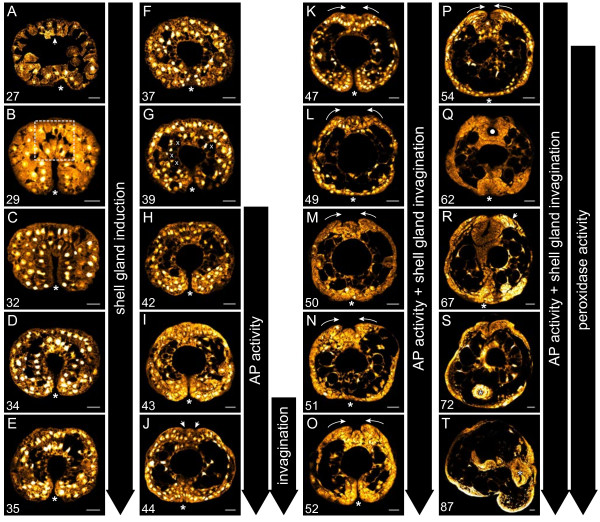
**Early shell development in *L. stagnalis *illustrated by Confocal Laser Scanning Microscopy (CSLM). A**-**F** During the course of gastrulation the initial invagination of the blastopore (asterisks) deepens to form the archenteron. The initial contact between dorsal ectoderm and endoderm is loose and is characterised by cellular projections (boxed region in **B**). **G**-**I** Upon contact, ectoderm and endoderm display signs of differentiation: the dorsal ectodermal cells at the contact zone differentiate into highly columnar shell field cells, and the endodermal cells are characterised by a lack of large vacuoles which are present in adjacent endodermal cells (indicated in **G** by white “x”s). **J**-**R** The initial bilateral invagination of the shell gland is visible in **J** (arrows). During this invagination the margins of the shell gland begin to converge (curved arrows in **K** to **P**). By 62 hpfc the non-invaginated margins of the shell gland have converged and form a closed lumen (white dot in **Q**). By 67 hpfc cells at the shell gland margin are highly elongated (arrow in **R**). Embryos in A-S are oriented with the shell field to the top and the veliger in **T** is oriented with the shell field to the left. An asterisk marks the position of the blastopore. Panels **A**-**P** are transverse optical sections and Panels **Q**-**T** are sagittal optical sections. All scale bars are 20 μm. Numbers in the lower left of each panel indicate the age in hours post first cleavage (hpfc).

Using CLSM we have studied the cell arrangements and movements of the embryo from early gastrulation (which precedes any contact between the dorsal ectoderm and the underlying endoderm) until evagination of the shell gland. At 27 hpfc the ventral ectoderm (opposite the future site of the shell field) is broadly depressed representing the initiation of gastrulation (Figures 1A’ and 2A). The proximal (‘basal’) side of the dorsal ectoderm faces inwards to a large blastocoel cavity (Figure 2A arrow). Between 29 hpfc and 32 hpfc the invagination of the archenteron initiates and completes. Cells of the archenteron assume an elongated shape from 29 to 35 hpfc (Figure 2B-E). By 30 hpfc in almost all embryos observed, the endodermal cells at the tip of the archenteron are in contact with the dorsal ectoderm. Initially, this contact does not exist over the entire surface of each cell. Rather, each endodermal cell appears to send out pseudopodia-like projections to the overlying ectoderm (Figure 2B boxed region). This results in small spaces being observed between the contacting cellular extensions. In other regions of the embryo, the cells of the invaginating archenteron are separated from the ectoderm by mesodermal cells or intercellular spaces.

The overall shape of the early 29 to 32 hpfc archenteron is slit-like (Figures 1B’ and 2B, C). Between 34 and 37 hpfc the blastopore opening narrows, and the archenteron develops a large round lumen (Figures 1C’ and 2D, E). In most embryos at this stage, a variable number of cells at the tip of the archenteron are in contact with dorsal ectodermal cells directly beneath the large head vesicle cells. By 37 hpfc the contact between endoderm and dorsal ectoderm appears to be firmly established (Figure 2F). This is the only region in the embryo where these two cell layers are in direct contact with each other, the archenteron is otherwise bordered by mesodermal cells or intercellular spaces. At this stage, neither the ectodermal nor the endodermal cells at the contact site display an altered cell morphology compared with their neighbours using Sytox Orange.

The first signs of differentiation of the dorsal ectoderm cells as observable by CSLM occur at 39 hpfc. At this stage, these cells take on a columnar morphology and are clearly distinguishable from adjacent ectoderm cells (Figure 2G). The columnar cells are in direct contact with four to five cells of the tip of the underlying archenteron. These endodermal cells in turn are characterised by a lack of large vacuoles that are present in adjacent cells of the archenteron (indicated by ‘x”s in Figure 2G). For this reason these archenteron-tip cells have been referred to as “small-celled entoderm” [28], here as small celled endoderm. The number of endodermal cells contacting presumptive shell gland cells remains low during the period of contact, never exceeding six cells. During the next hours the morphological differentiation of both cell layers becomes more pronounced with the nuclei of presumptive shell forming cells assuming a basal location (Figure 2I-K).

**Figure 3:**
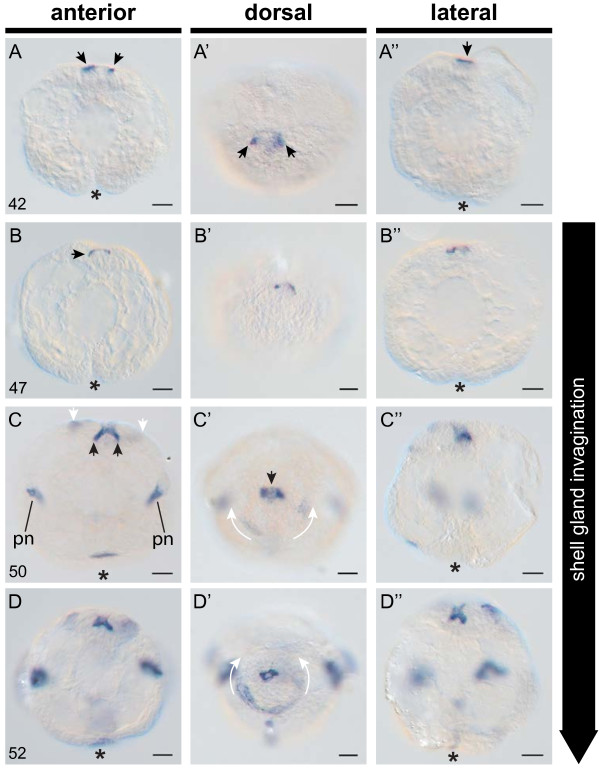
**Alkaline phosphatase (AP) activity in the early shell gland of *L. stagnalis*.** Endogenous alkaline phosphatase activity (dark blue precipitate in all panels) highlights the development of distinct cell populations and structures within the shell gland, and allows for the identification of distinct stages of larval shell development. **A**-**A”**(42 hpfc). The first evidence of AP activity in the shell gland occurs at 42 hpfc (arrows). **B**-**B”** (47 hpfc). Invagination of the shell gland begins at 47 hpfc. This is visible in AP^+^ cells which can be seen just below the outermost level of the dorsal ectoderm (arrow in **B**). **C**-**C”** (50 hpfc). Non-invaginated AP^+^ cells at the margin of the shell gland (white arrows in **C**) expand in an anterior direction (curved white arrows in **C’**). Invaginated AP^+^ cells (black arrows in **C** and **C’**) intensify their AP activity. The anlage of the protonephridia (pn) and apical plate also become AP^+^ at this stage. **D**-**D”** (52 hpfc). The non-invaginated AP^+^ cells at the margin of the shell gland continue to migrate in an anterior direction (curved white arrows in **D’**). All embryos are oriented with the shell field to the top. An asterisk marks the position of the blastopore. All scale bars are 20 μm. Numbers in the lower left of each panel row indicate the age in hours post first cleavage (hpfc).

**Figure 4:**
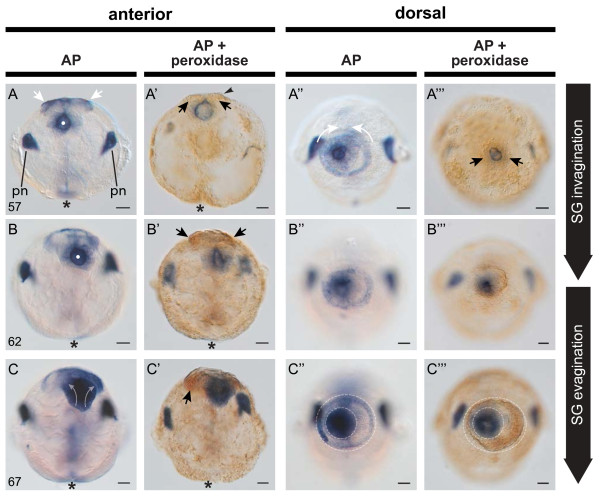
**Alkaline phosphatase (AP) and peroxidase (PO) activity in the mature shell gland of *L. stagnalis*.** Endogenous AP activity intensifies as the shell gland matures, and PO activity also becomes detectable. **A**-**A”’** (57 hpfc). The mature shell gland at 57 hpfc is characterised as a closed lumen with intense AP activity (white dot in **A**). AP activity has also increased in non-invaginated cells at the margin of the shell gland (white arrows in **A**), and in the anlage of the protonephridia (pn). Weak PO activity is also evident at this stage (black arrows in **A’** and **A”’**), and secreted insoluble material can also be seen in preparations of this age (arrowhead in **A’**). Non-invaginated AP^+^ cells at the margin of the shell gland finish their anterior expansion, meeting at the midline (curved white arrows in **A”**). **B**-**B”’** (62 hpfc). In 62 hpfc larvae, the shell gland is maintained as a closed lumen (white dot in ) while PO activity in non-invaginated cells intensifies (black arrows in **B’**). **C**-**C”’**(67 hpfc). Between 62 and 67 hpfc evagination of the shell gland has commenced (transparent white arrows in **C**) and PO activity in non-invaginated cells of the shell gland margin has increased (black arrow in **C’**). The asymmetry of the shell gland is made clearly visible by populations of AP^+^ and PO^+^ cells (dashed ovals in **C”** and **C”’** respectively). All embryos are oriented with the shell gland to the top. An asterisk marks the position of the blastopore. All scale bars are 20 μm. Numbers in the lower left of each panel row indicate the age in hours post first cleavage (hpfc).

The first external signs of shell gland differentiation are two lateral slit-like depressions that form directly beneath the large head vesicle cells at 42 hpfc (Figure 1D, D'). The first sign of endogenous AP activity can be detected at the same age in the two lateral depressions (Figure 3A), eight hours earlier than previously reported [17]. In transverse CSLM optical sections, the two lateral depressions of the dorsal ectodermare first observed at 44 hpfc and deepen in the following hours to form an invaginated shell gland (Figure 2J-Q). During the invagination process columnar cells at the periphery of the shell gland begin to converge towards each other (Figure 2K arrows). Between 50 hpfc and 52 hpfc the shell gland is comprised of two prominent lateral invaginations and a central elevation (Figure 2M-O). This bifurcated shell gland morphology is easily visualised by intense AP activity (Figure 3C, D). Scanning electron micrographs of this stage show a depression surrounded by a concentric arrangement of cells representing the non-invaginated part of the shell gland (Figure 1F). In the outer-most ring of cells, a second domain of AP activity can be detected which has not been previously reported for *Lymnaea*. This domain is first visible at 50 hpfc as a semicircle lining the posterior half of the shell gland (Figure 3C’, D’). During the next seven hours the tips of the semi-circle steadily extend anteriorly until a closed ring is formed (white arrows in Figure 3C’, D’ and 4A”'). From 54 hpfc onwards the central elevated part of the invagination flattens, and the non-invaginated shell gland margins continue to converge (arrows in Figure 2P). By 62 hpfc the margins have converged and the shell gland appears to be a sealed lumen (white dot in Figure 2Q). All invaginated cells of the shell gland at this stage are AP positive (white dot in Figure 4A, B), with AP activity in non-invaginated cells of the shell gland margin also persisting (white arrows in Figure 4A). At this stage, more than ten hours earlier than previously reported [17], the first signs of endogenous PO activity in and around the shell gland are evident. Peroxidase activity can be detected in non-invaginated cells directly adjacent to the shell gland lumen (arrows in Figure 4A’) and adjacent to the peripheral AP positive ring of cells (arrows in Figure 4A”’) which are not detected in double staining experiments against both enzyme’s activities. Also, between 52 hpfc and 57 hpfc, the first extra-cellular organic material has been secreted and is stretched over the entire shell gland (Figures 1F’ and 4A’ arrowhead). By 62 and 67 hpfc the non-invaginated cells at the periphery of the shell gland are highly elongated (arrow in Figure 2R). Scanning electron micrographs reveal the shell gland margin as an elevated ring (Figure 1H and I’). The secreted insoluble material now lies loosely on the elevated shell gland margin and displays PO activity (arrows in Figure 4B'). Peroxidase and AP activity also persists in adjacent non-invaginated rings (Figure 4B'' and B”’). The first asymmetry in the shell gland is also visible at 62 hpfc with the shell gland slightly shifted to the left side (indicated by the dashed ovals in Figure 1H’). This asymmetry becomes more pronounced in subsequent stages.

**Figure 5:**
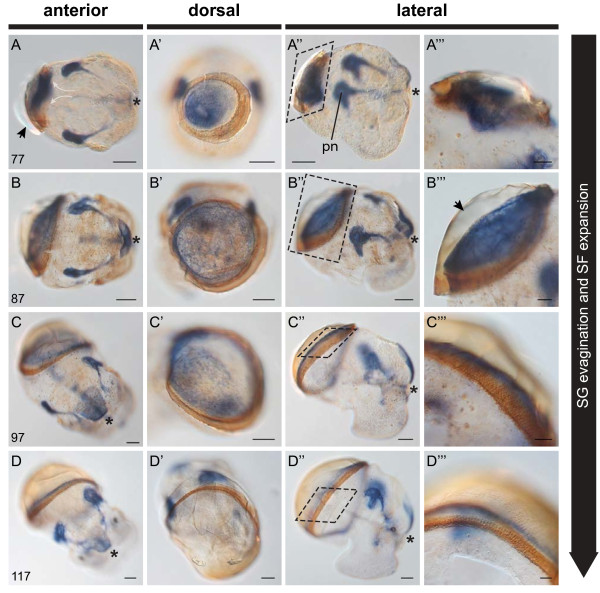
**Alkaline phosphatase (AP) and peroxidase (PO) activity in the evaginating shell gland and expanding shell field of *L. stagnalis *.** All panels are double labelled for AP and PO activity. **A**-**A”’** (77 hpfc). By 77 hpfc secreted birefringent material (black arrow in **A**) overlying the evaginating shell gland is clearly visible. The shell gland is no longer a closed lumen (cf. Figure 4A and 4B) as it continues to evaginate (indicated by transparent white arrows in **A**). A ring of intense PO activity now surrounds a field of AP activity (A’). The protonephridia (pn) remain intensely AP^+^ (**A”**). **B**-**B”’** (87 hpfc). By 87 hpfc shell gland evagination appears to be complete and the field of AP^+^ cells in the shell gland has expanded in all directions (**B’**). A sheet of organic material overlies the shell field (arrow in** B”’**). **C**-**C”’** (97 hpfc). At 97 hpfc the shell field continues to increase in size and the border between PO^+^ and PO^-^ cells at the shell field margin sharpens (C”’). **D**-**D”’** (117 hpfc). By 117 hpfc AP activity in the shell gland is concentrated in cells directly adjacent to PO^+^ cells. All larvae are oriented with the shell field to the left. An asterisk marks the position of the stomodaeum. Scale bars are 20 μm (**A”’**, **B”’**, **C”’** and **D”’**) or 50 μm (**A**-**A”**, **B**-**B**”, **C**-**C**” and **D**-**D”**). Numbers in the lower left of each panel row indicate the age in hours post first cleavage (hpfc). Panel A'' is reflected about the vertical axis for consistency of presentation.

**Figure 6:**
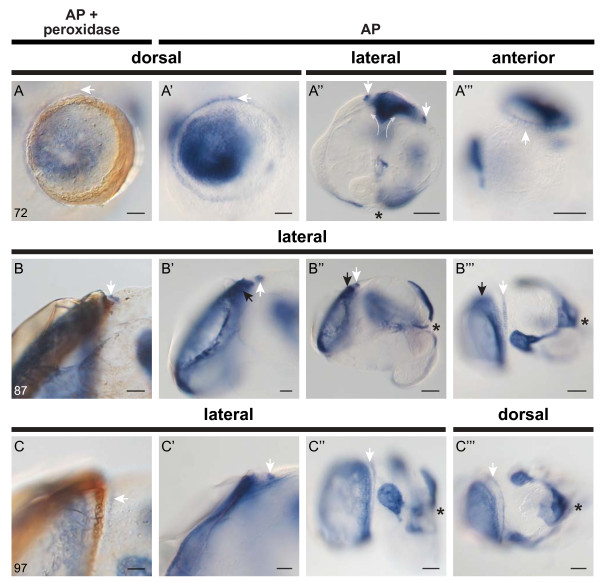
**Multiple alkaline phosphatase (AP) domains reveal a differentiated and complex organisation of the shell gland.** A second domain of AP activity present within the shell gland and shell field (not described by Timmermans [**17**]), is located outside of the PO domain and can not be clearly detected after prior detection of PO activity (see Figure 5 and Figure 6A cf. 6A'). **A**-**A'''** (72 hpfc). This second AP^+^ domain associated with non-invaginated cells of the shell gland margin can first be detected at 50 hpfc (see white arrow in Figure 3C), and is more pronounced at 72 hpfc (white arrows in **A**-**A'''**). This AP^+^ domain subsequently decreases in strength. **B**-**B”’** (87 hpfc). At 87 hpfc the distinct domains of AP activity associated with the evaginating shell gland (black arrows) and the non-invaginated cells of the shell gland margin (white arrows) are still visible. **C**-**C'''** (97 hpfc). By 97 hpfc the domain of AP activity associated with non-invaginated cells of the shell gland marginis considerably weaker (white arrows). It's position relative to PO^+^ cells can be seen clearly in **C** (white arrow). The larvae in **A**-**A”’** are oriented with the shell field to the top, the larvae in B-C”’ are oriented with the shell field to the left. An asterisk marks the position of the stomodaeum. Scale bars are 20 μm (**A**-**A’**, **B-B’** and **C**-**C’**) or 100 μm (**A”**-**A”’**, **B”**-**B”’** and** C”**-**C”’**). Numbers in the lower left of each panel row indicate the age in hours post first cleavage (hpfc). Panels **A”, A”’, B”,C** and **C”’** are reflected about the vertical axis for consistency of presentation.

From 67 hpfc onwards the shell gland evaginates giving rise to the shell field. First, the non-invaginated margins of the shell gland diverge, opening up the shell gland lumen (Figure 2S and curved arrows in Figure 4C, 5A and 6A). Contact between endodermal cells and the dorsal ectodermal is lost at 77 hpfc, and the shell field expands in size during subsequent development. Peripheral cells (formerly non-invaginated cells of the shell gland) maintain their columnar shape whereas the central, formerly invaginated cells flatten (Figure 2T).

The relative arrangement of PO and AP activity domains persists from 77 hpfc to 117 hpfc. The centre of the shell field displays AP activity with increasing intensity towards the shell field margin (Figure 5C, C’, D”). During the following stages AP activity in the centre of the shell field gradually decreases (Figure 5C, D). At 117 hpfc AP activity is found in a line of cells proximal to the shell field margin (Figure 5D). At this stage the secreted organic material and the highly elongated cells of the shell field margin continue to exhibit a strong PO activity (Figure 5C”’ and D”’).

In the periphery of the PO positive shell field margin, a faint ring of AP^+^ cells is detectable from 72 hpfc on (Figure 6). This signal possibly represents the AP^+^ domain of non-invaginated cells seen in earlier stages (see white arrows in Figures 3 and 4). During the course of shell field differentiation, the activities of both enzymes are continuously located in adjacent, non-overlapping cell populations within the shell field (Figure 6A-C”’; see Figure 8 for a schematic summary of these observations).

### Non-shell related AP and PO activities during larval development

**Figure 7:**
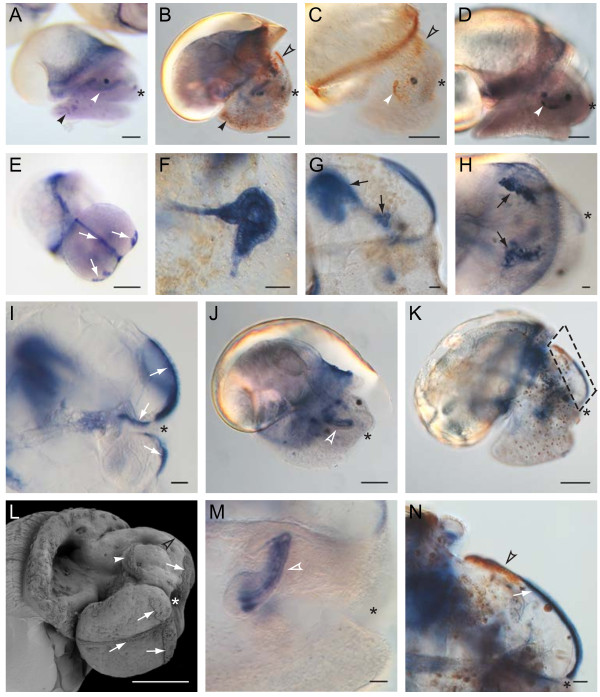
**Endogenous alkaline phosphatase (AP) and peroxidase (PO) activities as markers of larval development.** Other organs and cell populations not involved in shell formation display AP and PO activity. Endogenous AP activity can be found in most ciliated fields (**E** and **I**, white arrows), including the apical plate (**G**, **I**, **K**, **N**), the protonephridia (**F**-**H**, black arrows), the developing radula (**J** and **M**, white open arrowhead) and dispersed cells throughout the foot (A, black arrowhead). Peroxidase positive structures include the head vesicle cells (**B**, **C**, **K** and **N**, black open arrowheads), the velum (**C**, white arrowhead) and scattered cells throughout the foot and head tissue (**B**, black arrowhead, and **K** respectively**)**. N is a detailed view of the boxed area in K. **L** is an SEM from a lateral/ventral perspective of a 5+ dpfc staged larva illustrating the ciliated fields of the foot and head (white arrows) and the velum (white arrow head). **A**-**D**, **H**, **J** and **M** show larvae of 5+dpfc, **F** shows a 77 hpfc old larva, I shows a 97 hpfc old larva and **E**, **G**, **K** and **N** show 117 hpfc old larvae. The position of the stomodaeum is marked by an asterisk. Scale bars are 20 μm (**F**-**I**, **M** and **N**) or 100 μm (**A**-**E**, **J**, **K**, **L**). Panels **B**, **G**, **I**, **K**, and N are reflected about the vertical axis for clarity of presentation.

Endogenous AP and PO activities can also be used to follow the development of larval structures in *L. stagnalis* in a more general way. Several structures besides the shell gland and shell field display endogenous activity of these enzymes (Figure 7). AP activity is present in most ciliated fields (Figure 7E, I, K and N) and the protonephridia (Figure 7F-H) [26]. In older 5+ dpfc stages the developing radula (Figure 7J and M) and cells throughout the foot exhibit AP activity (Figure 7A). Endogenous PO activity can be found in ectodermally derived cells scattered over the head and foot region (Figure 7B and K) and later in the head vesicle cells anterior from of the apical plate (Figure 7B, C, K and N) [33]. Both enzymes show adjacent, but non-overlapping activity in the head region and activity in the velum [33] (Figure 7C and D).

## Discussion

In various molluscan groups, the initial differentiation of the dorsal shell forming ectoderm has been observed to coincide with the presence of a tight contact with underlying endodermal cells (reviewed in [11]). These observations raised the possibility that this contact is required for the specification of molluscan shell forming cells in general. Based on manipulative experiments, first Raven [26] and later Hess [34,35] concluded that it is this dorsal ectoderm/endoderm contact that specifies the shell field. Raven differentiates between two possibilities of contact-dependent shell field specification, induction vs. activation. Raven [26] realised that if the dorsal ectoderm is truly induced to become the shell gland by such a contact (rather than activated as would be the case if a population of dorsal ectodermal cells were already specified in someway), two morphogenic preconditions must be realised. Firstly, only a restricted part of the endoderm (i.e. cells at the tip of the archenteron) should be able to elicit this specification in the overlying ectoderm. Secondly, the whole ectoderm (or at least the majority) should be able to respond to this induction by forming a shell gland. Raven’s observations based on four embryos (whose gastrulation had been perturbed by lithium and which developed an ectopic shell gland) lead him to conclude that most of the ectoderm is indeed able to respond to signals from the underlying endoderm, and that therefore the dorsal ectoderm is truly induced by the endoderm to become shell forming tissue, rather than activated. This model of induction-mediated shell field specification has since been supported [34], modified [13,35] and contradicted [36-38]. These studies report a wide capacity of the ectoderm to form a shell field, but disagree about the origin of the “inductive cue”. Hess’s observations of partial embryos after blastomere separation in *Bythinia* and *L. stagnalis*[34,35] support the contact dependent model of shell field specification, but indicate that any endodermal tissue, even single cells, is capable of induction. Cell deletion experiments in *Ilyanassa*[13] support the hypothesis that there is no cellular specificity in the inducing endoderm: all combinations of ectoderm and endoderm can generate a shell field. Furthermore, in *Ilyanassa* it has been shown that the tip of the archenteron is never in close proximity to the dorsal ectoderm [37]. Labordus and van der Wal [38] extending the studies on *Ilyanassa* by Clement [36] and Cather [13], suggest a scenario which distinguishes between the histogenic and morphogenic differentiation of the shell gland. Based on observations of misdeveloped embryos producing internal shell material, Labordus and van der Wal [38] propose that the histogenic differentiation necessary to produce such material is independent of inductive interactions, whereas the correct spatial organisation of shell forming tissues depends on spatially correct inductive interactions between the D-quadrant macromeres and the overlying micromeres earlier in development. Based on that study, McCain [39] conducted cell deletion experiments that suggest inductive interactions among the micromeres are also required to give rise to the larval shell forming tissues. The disturbance of these interactions by the removal of participating cells leads to the internal deposition of calcium carbonate similar to those observed by earlier workers, supporting the assumption that the processes leading to the histogenetic vs. the morphogenetic differentiation of larval shell forming tissues do not depend on each other. This hypothesis is further corroborated by work in experimental systems that allow for an artificially induced shell internalisation, e.g. by exposure to environmental toxins such as platinum [40,41]. In these systems (*Marisa cornuarietis* and *Planorbis corneus*), platinum interferes with the localisation of shell material and the growth of shell forming tissues, while the cellular differentiation of these tissues appears to remain unaffected [40,41].

While Raven’s hypothesis of induction-mediated shell gland specification still represents the most comprehensive theory of how the future shell forming cells are initially specified in molluscs, contradictory observations have been reported for a number of disparate taxa. In the pulmonate taxa *Bradybaena* and *Achatina marginata* the shell field is differentiated before any contact with the underlying endoderm is established, in the Caenogastropod *Marisa* this contact is interrupted by intermingled cells, and in other gastropod species (*Ilyanassaobsoleta* and *Achatina fulica*) as well as bivalves (*Cyclas* and *Sphaerium*) no contact is present (reviewed in [11]). Unfortunately, none of these previous studies utilised high resolution imaging techniques such as CLSM that are available today, or conducted their investigations with high temporal resolution. Nonetheless we must acknowledge that there does exist the possibility of an alternative, contact-independent shell gland specification mechanism, leaving open the question as to whether the dorsal ectoderm/endoderm contact event represents the ancestral molluscan mode of shell gland specification.

While the present study was not intended to clarify the molecular mechanisms of shell gland specification, nor to differentiate between scenarios of induction vs. activation as proposed by Raven [26], it does clarify the nature of the cellular interactions between endoderm and ectoderm prior to and during shell gland specification, and also provides an accurate framework for the timing of these events in *L. stagnalis* at 25°C. Using CSLM we could reconstruct the cellular arrangements and movements during contact between dorsal ectoderm and endoderm. Despite its importance, former studies do not include a description of how this contact is initially established. If, as Raven [26] proposes, the ‘small-celled endoderm’ truly induces the overlying dorsal ectoderm in *Lymnaea*, these endodermal cells should have acquired their inductive capacity prior to contact. Such prior differentiation is not revealed by Sytox Orange staining in our study (both dorsal ectoderm and the ‘small-celled endoderm’ show the first signs of differentiation after contact establishment; see Figure 2F and G), nor by Raven using standard histological stains [26]. Raven concluded that acquisition of an inductive capacity by the endoderm is not revealed by any histological differentiation. Indeed, a molecular differentiation of the contacting endoderm could be expected to precede any visible histological differentiation. Identification of such molecular markers would provide great insight into the evolution of the molluscan shell.

Our study also reveals a pronounced bilateral organisation of early shell gland development; the invagination of the shell gland begins when two lateral points of the thickened dorsal ectoderm form two lateral depressions (Figure 1D-E’, 2J-O). This bilateral organisation persists until the margins of the shell gland converge above the lumen of the invaginated shell gland and the bifurcated lumen rounds up (Figures 1F’, 2P-R). The formation of this shell gland lumen coincides with the secretion of the first insoluble shell material. None of the invaginated shell gland cells appear to participate in the secretion of this first water insoluble material which emerges from the peripheral non-invaginated shell gland cells [27] (Figure 1F’). This observation has raised the hypothesis that the process of shell gland invagination is required in order to bring cells at the periphery of the shell gland into close contact, and to thereby initiate the secretion of an insoluble shell forming matrix without a central hole above the shell gland lumen [42,43]. Shortly afterwards, the first signs of asymmetry in the shell gland appear. The invaginated part of the shell gland shifts to the left side which generates a larger distance between the centre of the lumen and the peripheral secreting cells on the right side than on the left side (Figures 1H’ , 4C” , C”’). This early asymmetry presumably reflects the future coiling direction of the mature shell.

While shell gland formation is a deeply conserved feature of molluscan development, there is considerable diversity in its ontogeny within and between all molluscan groups. For those species with internal or reduced shells, the formation and further differentiation of the shell gland differs from that seen in *L. stagnalis* and other externally shelled molluscs (reviewed in [11]). In shell-less cephalopods for example, shell gland development ceases during dorsal ectoderm invagination, and an evaginated shell field never forms. In cephalopods with an internal shell, the shell gland is internalised and characterised by a closed pore, and is therefore referred to as a “shell sac”. This structure is not thought to be formed by an invagination of the central part of the thickened dorsal ectoderm. Instead, the peripheral cells of the thickened dorsal ectoderm bulge upwards and overgrow the central cells. An internalised shell gland or “shell sac” is also found in shell-less terrestrial slugs, but is formed by a different mechanism. Here, the dorsal ectoderm invaginates as it does in shelled gastropods, but continues inwards leading to a complete internalisation of all shell gland cells, and consequently a closure of the shell gland. These ontogenetic events in secondarily shell-less slugs also illustrate their common ancestry with shelled snails such as *Lymnaea*.

### Endogenous enzyme activities as a tool to illustrate larval development

**Figure 8:**
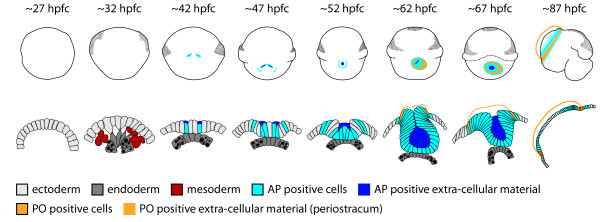
**A schematic representation of the major events during early development of the shell gland and shell field in *L. stagnalis*.** Uppermost row are all dorsal views except for ~87 hpfc which is a lateral view. Lowermost row are the corresponding transverse sections through the developing shell gland and shell field. By approximately 32 hours post first cleavage (hpfc) endodermal cells at the tip of the archenteron, the so called“small celled endoderm” for their lack of large vacuoles (indicated by black ovals and circles), have made contact with the overlying cells of the dorsal ectoderm. These are the only endodermal cells to make contact with ectoderm. By 42 hpfc cells that are in contact with the endoderm have thickened and some cells display alkaline phosphatase (AP) activity. The strongest AP activity is apparently in extra-cellular material. A bilateral invagination of the shell gland has also commenced at this age. By 47 hpfc the bilateral invagination has deepened and non-invaginated, posterior shell gland cells at the periphery of the shell gland also display AP activity. At 52 hpfc the bilateral invaginations have fused, and the lumen of the shell gland displays intense AP activity. By 62 hpfc the lumen of the shell gland appears to be sealed with intense AP activity. The first peroxidase (PO) activity is visible at this time and is evident in cells and in the secreted periostracum material. By 87 hpfc the form of the juvenile snail has been established and the non-overlapping zones of AP and PO activity are maintained.

Our study demonstrates the usefulness of endogenous AP and PO activity as markers to map molluscan development. During the course of shell gland and shell field differentiation both enzymes are continuously located in distinct shell forming cells (summarised in Figure 8). Larval structures such as ciliated fields and the protonephridia are also AP positive, and the head vesicles in older embryos display PO activity (Figure 7). Previous studies of AP activity during shell field development in *L. stagnalis* were based on acetone-fixed and paraffin-embedded sections, a procedure that results in a significant loss of enzyme activity [17]. Using the methods we describe here, we can detect both earlier and novel domains of AP and PO activity, and can also simultaneously detect AP and PO activities. AP and PO activity in shell forming tissues has been shown for a number of gastropod and bivalve taxa (summarised in [17]). Transcripts encoding these enzyme families derived from developmental stages and/or adult shell-secreting tissues can be found in sequence databases for divergent molluscan taxa, suggesting that these enzymes might have conserved functions during molluscan shell development. While the precise function of AP in molluscan shell forming tissues has not been described, AP activity in vertebrate bone (hydroxy apatite) forming tissues is known to regulate levels of inorganic pyrophosphate, a potent inhibitor of mineralisation [44,45]. PO activity is displayed by non-invaginated cells at the periphery of the shell gland. These cells appear to be intimately associated with the production of the periostracum which is itself PO^+^ (Figures 4, 5, 6). It has been suggested that peroxidases in the periostracum may assist in the crosslinking of periostracal proteins, rendering them insoluble and resistant to abrasion [17,27]. These simple histochemical assays provide a tool not only to identify and trace functionally distinct cell populations within the developing shell gland and shell field, but also to simply assist with the orientation of the molluscan embryo. In the future, such assays could be used to assess the effects of manipulative experiments such as shell-specific gene knock-down assays.

## Conclusions

This work represents a platform from which analyses aimed at the identification of the molecular regulators responsible for shell development in *L. stagnalis* can be conducted. We have described the timing of developmental events critical to specification of shell forming cells, and the movements of cells that take part in these processes. We also highlight the use of histochemical assays that allow for the detection of endogenous alkaline phosphatase and peroxidase activity within shell forming cells. Understanding the molecular basis of shell development from a range of molluscan representatives will provide deep insight into the evolutionary events that supported the generation of much of today’s molluscan diversity. The work presented here is a first step towards the development of *L. stagnalis* as a model to understand how this diversity arose.

## Competing interests

The authors declare that they have no competing interests.

## Author’s contributions

JH carried out the experimental procedures. DJJ conceived and supervised the study. Both authors participated in the design of the study and drafted the manuscript. Both authors read and approved the final manuscript.
